# Do intangible factors enhance sociocultural productivity and economy in world heritage sites?

**DOI:** 10.3389/fpsyg.2024.1393811

**Published:** 2024-05-31

**Authors:** María Martín-Lucas, Ana Leal-Solís, Ángel Pizarro Polo, Rafael Robina Ramírez, Libertad Moreno-Luna

**Affiliations:** ^1^Department of Business Management and Sociology, Universidad de Extremadura, Cáceres, Spain; ^2^Department of Building, Universidad de Extremadura, Cáceres, Spain

**Keywords:** productivity, heritage sites, tourism, emotions, architecture, economy

## Abstract

Measuring the sociocultural productivity of heritage sites remains an ongoing issue for international organizations concerned with the conservation and promotion of traditional sites. The productivity of these locations is not only affected by tangible elements but also by intangible factors, such as the emotions generated by the experiences. For this purpose, 597 employees of hotels in these historical locations who had visited one of the 14 heritage sites in Spain assessed what role emotions play in this contribution. The methodology used was the application of structural equations. Several conclusions have been drawn utilizing the SmartPLS 4 software. The first is that the generation of positive emotions comes exclusively from cultural and historical dynamization and not from technological advances or an eagerness to learn. The second is that both the application of technological advances and cultural dynamization have a direct impact on productivity.

## 1 Introduction

Heritage sites, renowned for their cultural, historical, architectural and natural significance, attract visitors from all areas of the world (Sites, 2008; Loureiro et al., 2022). They not only contribute to local economies through tourism and cultural exchange (Cochrane and Tapper, 2006), but also define part of the productivity of urban spaces (Rypkema, 2008; Campoy-Muñoz et al., 2017).

The factors that explain the productivity of urban areas remain difficult to measure and identify (Lobo et al., 2011; Yang et al., 2022). These factors depend on urban development models. Some urban models evaluate the level of productivity in terms of the economic density that such areas maintain (Ahlfeldt and Pietrostefani, 2017). Others measure productivity in urban areas in cost reductions from improved communications (Glaeser, 2010) and the development of innovation in firms (Quigley, 1998).

Sociocultural productivity in heritage site is directly connected to their designation as sites of cultural, historical, architectural scientific or natural heritage for their unique and significant value (Flores de León et al., 2020). This heritage is not only tangible but also intangible, based on mythologies, and is a resource for the present (Su, 2020; Ranwa, 2022).

This new approach to productivity in historic sites brings with it an added difficulty of balancing conservation and urban development (Puren and Jordaan, 2014; Loureiro et al., 2022; Yang et al., 2022). This balance is put at risk by the constant tourism challenges to which traditional sites are subjected (United Nations Educational, Scientific and Cultural Organization, 2020; United Nations World Tourism Organization, 2020).

This challenge involves investing in the education and cultural, historical or religious experiences (McKercher, 2002; Robina Ramírez and Pulido Fernández, 2018; Yang et al., 2022). These experiences generate emotions as a form of knowledge (D’Hauteserre, 2015).

However, how these emotions impact the productivity of historic environments has not yet been determined (Zhu et al., 2022). Emotions are significant drivers of heritage tourism experiences (Medina-Viruel et al., 2019); how heritage tourism elicits positive (joy, happiness and pleasure) and negative (e.g., guilt, sadness and regret) emotions needs further research (Yang et al., 2022; Prayag and Del Chiappa, 2023).

The role that emotions play in enhancing sociocultural productivity is also affected by several sociocultural factors. 1. The application of technological advances to maintain the integrity of historic environments through multimedia installations, narratives and interactive exhibits (Song and Selim, 2022). 2. Historical and cultural invigoration through: organizing cultural events, festivals and performances (Ebejer, 2019). 3. Community educational engagement triggering positive emotions associated with learning and discovery (Fan, 2014).

## 2 Literature review

### 2.1 The socio-cultural productivity of world heritage sites (P)

Due to the importance of the tourism sector, international agencies have placed a high value on productivity analysis (Sliem et al., 2019; Fu et al., 2023). Urban World Heritage Sites (WHS) have immense cultural, historical, architectural and economic importance and effectively contribute to local economies (Bowitz and Ibenholt, 2009). Economic productivity has been widely studied in the simplest dimension and measured through the number of visitors, tourism income, job creation and contribution to the local economy (Loulanski, 2006a,b; Petronela, 2016; Parte and Alberca, 2023). Sociocultural productivity, in the context of urban WHS, goes beyond mere economic production (Caust and Vecco, 2017).

The cultural and social productivity of an urban WHS is related to its ability to foster a sense of identity, pride and social cohesion among local communities (Tan and Tan, 2020; Zhou et al., 2022; Folgado-Fernández et al., 2024). To measure this dimension, some studies have included the participation of local residents in cultural activities, educational programmes and community events related to the heritage site. In those studies, are measured elements like (1) community engagement and participation, the active involvement of local residents in cultural activities, educational programs, and community events related to the heritage site is essential for fostering socio-cultural productivity, (2) heritage interpretation and education through programs to enhance socio-cultural productivity by promoting understanding, appreciation, and stewardship of cultural heritage among local communities, (3) cultural diversity and inclusivity, it thrives in environments that embrace cultural diversity and inclusivity, where different perspectives, traditions, and identities are celebrated and respected, (4) social cohesion and collaboration among local stakeholders. They are fundamental for sustaining socio-cultural productivity within an urban WHS, (5) economic sustainability and livelihoods to support socio-cultural productivity by providing opportunities for local residents to derive economic benefits from heritage-related activities and businesses (Parga-Dans et al., 2020).

This socio-cultural participation can be extended from residents to the parties that interact in the WHS, such as the local tourism administration, companies on the supply side and tourists (Moli, 2011; Al-Hammadi, 2022). This interaction makes it possible to develop sociocultural tourism products that can be measured (McCamley, 2016; Cheng and Chen, 2022). Sociocultural products also contribute to developing an identity, culture and heritage based on the historical and cultural revitalisation of said ancient spaces (Nocca, 2017; Al-Hammadi, 2022).

### 2.2 The value of emotions (E)

The study of the emotions generated by the experiences of employees working in heritage places offers very relevant information for the analysis of the productivity of these spaces (Abd Aziz et al., 2020). Hotel employees are first-hand witnesses to the day-to-day experiences at heritage sites and can provide more qualified information than tourists themselves by knowing how the attractiveness of these places’ impacts on life within these historic spaces (Robina-Ramírez et al., 2023a,b). Although emotions play an important role in the tourism experience, rigorous empirical research on this topic is limited (Abd Aziz et al., 2020; Hosany et al., 2020; Robina-Ramírez et al., 2023a). As Timothy (2011) noted, we know relatively little about the psychological and emotional underpinnings that drive WHS visitation.

According to cognitive appraisal theory, emotions are mental states that result from the processing or evaluation of personally relevant information (Roseman et al., 1990; Robina-Ramírez et al., 2020). In particular, goal congruence as evaluation determines the emotional response (Hosany et al., 2020). Furthermore, several studies argue that a location’s WHS status is an important aspect of its attractiveness (Nguyen and Cheung, 2014), but increased visitation as a result of being listed as a WHS can have negative impacts on the site’s sustainability (Tarawneh and Wray, 2017; Zhang et al., 2020).

Cultural, historical and natural treasures are not mere physical landmarks, but repositories of stories and traditions that have shaped human civilisation by connecting visitors to their origins (Tung and Ritchie, 2011; Abd Aziz et al., 2020). When visitors explore venerable temples or ancient ruins, they may feel a sense of awe and reverence for the people who built these places (Salemink et al., 2020), beauty and joy (Nisbet and Zelenski, 2011).

Urban heritage contributes significantly to the cultural identity of a city (Zhang et al., 2020). For locals and tourists alike, these places often evoke feelings of belonging and pride (Abd Aziz et al., 2020; Butler et al., 2022; Folgado-Fernández et al., 2024). Locals may experience a deep sense of attachment, as their personal histories become intertwined with the city’s history generating empathy and connection related to the achievements of past generations (Lang et al., 2023). This intertwining can give rise to a sense of nostalgia from the collective memories embedded in the architecture, streets and landmarks (Prayag and Del Chiappa, 2023).

Fostering positive emotional connections can create a virtuous circle that benefits both visitors and workers at the sites themselves (Van Dijk and Kirk, 2008; Veetikazhi et al., 2023). When tourists connect emotionally with a site, they become advocates for its protection (Fang et al., 2021). This emotional investment can lead to increased awareness of the importance of sustainable tourism practices, ensuring that these sites remain intact for future generations (Mugobi and Mlozi, 2021).

Based on what has been indicated so far, we can formulate hypothesis 1 (H1):

*H1:* E positively affect P.

### 2.3 Historical and cultural dynamisation (DHC)

Heritage sites are invaluable repositories of the evolution of human civilisation (Loulanski, 2006a,b; Arumugam et al., 2023). These sites are not mere relics frozen in time, but dynamic entities that reflect the constantly changing interaction between culture, history, architecture and society (Hede, 2008; Mugobi and Mlozi, 2021).

Heritage sites encapsulate moments that shaped societies and civilisations, allowing future generations to glimpse a distant era (Lowenthal, 2005; Zhu et al., 2022; Arumugam et al., 2023). By maintaining these physical links to the past, heritage sites help individuals to better understand origins, achievements and challenges (Rouhi, 2017; Cheng and Chen, 2022). These sites embody cultural practices, traditions and values that have been passed down through the generations (Vecco, 2010; Yang et al., 2022).

Local communities and societies often derive a sense of pride and belonging from these sites (Jimura, 2018; Fu et al., 2023), facilitating interactions between diverse communities and fostering a greater appreciation of different cultures (Su and Wall, 2011; Cheng and Chen, 2022). This cultural exchange enriches societies by broadening perspectives and promoting tolerance (Musitelli, 2002; Yang and Wall, 2022). The attractiveness of heritage sites has a significant impact on tourism, which in turn contributes to socio-economic development (Chong and Balasingam, 2019; Arumugam et al., 2023). The two hypotheses we can formulate are:

*H2:* DHC positively affects E.

*H3:* DHC positively affects educational dynamisation (DE).

### 2.4 Educational dynamisation (DE)

In recent years, there has been a growing interest in promoting responsible tourism and fostering a deeper understanding of these sites among local visitors (Leslie, 2012; Rasoolimanesh et al., 2021). To achieve this goal, tourism authorities play a crucial role in providing educational training programmes that enhance the experience of local visitors at heritage sites (Ritchie, 2003; Abd Aziz et al., 2020; Robina Ramírez and Fernández Portillo, 2020).

Educational training programmes organised by tourism authorities at heritage sites provide opportunities for local visitors to connect with their own history and cultural identity (Timothy, 2011; Rasoolimanesh et al., 2022). Those programmes bridge this gap by providing knowledge beyond what is on display (Fyall et al., 2020).

In preserving these sites, tourism authorities have a responsibility to promote responsible tourism practices among local visitors protecting the heritage sites of mass tourism (Pedersen, 2002; Leal-Solís and Robina-Ramírez, 2022).

An enhanced visitor experience is at the heart of educational training programmes making heritage sites come alive for local visitors (Alazaizeh et al., 2019). The successful implementation of educational training programmes requires collaboration between tourism authorities, local communities, historians, archaeologists and educators. Based on the above, the following hypotheses can be formulated:

*H4:* DE positively affects E.

*H5:* DE positively affects P.

### 2.5 The development of technological advances (T)

To date, numerous studies have addressed the role of technology in urban areas by identifying new business opportunities (Koukopoulos et al., 2017; Graziano and Privitera, 2020; Yan and Wall, 2022).

Heritage places do not only seek a consolidated, accessible and safe destination positioning (Santa-Cruz and López-Guzmán, 2017). In the continuous effort to improve customer engagement, the incorporation of technology is beginning to play an important role in improving visitor perception (Shafiee et al., 2022).

In parallel, research on interactive technology design for the cultural sector has also shifted its focus to implementing digital forms to provide means of dialogue and community engagement around heritage (Ciolfi et al., 2015; Yan and Wall, 2022). The quest for greater community engagement in the heritage sector has been encouraged by new possibilities offered by the advancement of digital technologies (Affleck and Kvan, 2008; Liang et al., 2021). In this scenario, cultural institutions seek to increase audience engagement with their collections and foster dialogue with their visitors, adopting more audience-centred practices (Simon, 2010; Mugobi and Mlozi, 2021).

Technology co-design methodologies have also contributed to empowering cultural heritage professionals to enhance visitor experiences (Ciolfi et al., 2016; Yan and Wall, 2022). In addition to the impact on heritage professionals, digital technologies are also having a significant impact in terms of supporting community participation and engagement in the cultural sector (Giaccardi, 2012). As a result, we have witnessed a proliferation of community-led cultural heritage initiatives that leverage platform solutions to manage cultural heritage (Giglitto, 2017). The hypotheses to be explored are:

*H6:* T positively affects DHC.

*H7:* T positively affects E.

*H8:* T positively affects P.

## 3 Methodology

In our quest to delve into the socio-cultural productivity of World Heritage Sites (WHS), we meticulously engaged with two primary sources of knowledge: firstly, the tourism authorities of the 14 territories hosting heritage sites in Spain; and secondly, the tourism companies operating within the historical destinations of these cities, particularly the hotels nestled within the heritage sites.

To ensure the robustness of our research, we initiated a pre-test phase to scrutinize the validity of variables crucial to socio-cultural productivity in heritage destinations. Communication with senior personnel within the tourism structure via email facilitated collaboration from all 14 territories, providing us with updated contacts of their managerial staff overseeing hotels within the heritage sites.

Moreover, to tailor the indicators to our research scope, three focus groups were conducted, involving 27 hotel employees. The insights gleaned from these sessions were invaluable in refining our questionnaire, a process overseen by the “ethical clearance” protocol, as per the guidelines outlined in document 18–2023 by the University of Extremadura.

The resulting online questionnaire comprised a succinct introduction and three distinct segments. The first segment delved into factors measuring WHS productivity, while the second segment explored indicators associated with experiences arising from technological advancements, historical, cultural, and educational dynamism. The third segment concluded with socio-demographic data, providing comprehensive insights into respondent profiles. These segments were meticulously crafted to ensure relevance and depth, with detailed indicators outlined in Table 1.

**Table 1:** Constructs indicadors.

Constructs/ Indicators	Variables	Authors
**P**	**Sociocultural productivity in heritage sites**	
P1	Sociocultural productivity measures the ability to foster a sense of identity, pride, and social cohesion among local communities	Tan and Tan (2020), Zhou et al. (2022), and Folgado-Fernández et al. (2024)
P2	Sociocultural productivity measures the participation of local residents in cultural activities, educational programs and community events related to the heritage site	Parga-Dans et al. (2020)
P3	The measurement of sociocultural productivity should broaden the interactions between the local tourism administration, companies on the supply side and tourists.	Moli (2011) and Al-Hammadi (2022)
P4	The interaction between the agents that intervene in the WHS allows the development of sociocultural tourism products that can be measured	McCamley (2016) and Cheng and Chen (2022)
**E**	**Emotions**	
E1	Awe, beauty and reverence for the people who built and inhabited these places.	Nisbet and Zelenski (2011) and Salemink et al. (2020)
E2	When tourists immerse themselves in local customs, traditions and ways of life they experience joy, empathy and connection.	Lang et al. (2023)
E3	Urban heritage contributes to a city’s cultural identity, evoking feelings of belonging, pride and empathy.	Butler et al. (2022)
E4	Locals can experience a deep sense of attachment and nostalgia, as their personal stories become intertwined with the city’s history.	Prayag and Del Chiappa (2023)
**T**	**Technology**	
T1	The incorporation of technology plays an important role in improving visitor perception.	Shafiee et al. (2022)
T2	Technology empowers cultural heritage practitioners	Ciolfi et al. (2015) and Yan and Wall (2022)
T3	Technology facilitates the pursuit of greater community engagement in the heritage sector	Affleck and Kvan (2008) and Liang et al. (2021)
T4	Technology encourages the fostering of dialogue with its visitors, adopting more audience-centred practices.	Simon (2010) and Mugobi and Mlozi (2021)
**DHC**	**Historical and cultural dynamisation**	
DHC 1	Heritage places reflect the constantly changing interaction between culture, history and society.	Hede (2008) and Mugobi and Mlozi (2021)
DHC 2	Historical and cultural revitalisation gives future generations a glimpse of a bygone era.	Lowenthal (2005), Zhu et al. (2022), and Arumugam et al. (2023)
DHC 3	Historical and cultural revitalisation helps to better understand the origins by fostering shared identity.	Rouhi (2017) and Cheng and Chen (2022)
DHC 4	Historical and cultural revitalisation leads to the preservation and continuation of customs.	Jimura (2018) and Fu et al. (2023)
DHC 5	Historical and cultural revitalisation fosters cooperation and understanding between people of diverse backgrounds.	Musitelli (2002) and Yang and Wall (2022)
DHC 6	Historical and cultural revitalisation sites inject income into local economies.	Chong and Balasingam (2019) and Arumugam et al. (2023)
**DE**	**Educational revitalisation**	
DE1	Educational revitalisation helps to foster a deeper understanding of these sites among local visitors.	Leslie (2012) and Rasoolimanesh et al. (2021)
DE2	Tourism authorities should provide educational training programmes that enhance the experience of local visitors.	Ritchie (2003) and Abd Aziz et al. (2020)
DE3	Educational training programmes help to better connect with cultural history and identity.	Timothy (2011), Rasoolimanesh et al. (2022), and Rasoolimanesh et al. (2021)
DE4	Educational revitalisation provides knowledge beyond what is on display	Fyall et al. (2020)
DE5	Educational training programmes have an impact on the conservation and protection of heritage sites.	Pedersen (2002) and Leal-Solís and Robina-Ramírez (2022)
DE6	Educational training programmes help heritage sites come alive for local visitors.	Alazaizeh et al. (2019)

### 3.1 Sample design and data collection

In our meticulous data collection process, we cast a wide net, engaging with hotel managers and employees across various departments in Spain through an online questionnaire. Collaboration with tourist agencies in close proximity to heritage sites facilitated engagement with a broad spectrum of establishments. Information was disseminated to 114 hotels, yielding 62 affirmative responses. Further refinement ensued, with 51 hotels meticulously selected based on information garnered from tourist agencies located near the 14 heritage cities. These establishments served as the bedrock for data collection, from which 597 valid questionnaires were obtained, meticulously ensuring the integrity and robustness of our dataset (see Table 2).

**Table 2:** Population and sample.

Spanish heritage cities accredited by ICOMOS	Hotels	Hotel sample	Number of employees sample
Historic Centre of Cordoba	10	6	71
Old town of Segovia and its aqueduct	7	4	36
Old town of Santiago de Compostela	12	7	30
Old town of Avila and its churches outside the city walls	7	2	24
Old city of Cáceres	4	3	49
Historic city of Toledo	11	6	51
Old city of Salamanca	9	3	77
Archaeological ensemble of Mérida	6	2	35
Historic city walled city of Cuenca	7	3	26
University and old town of Alcalá de Henares	6	3	30
San Cristóbal de la Laguna	8	3	29
Archaeological Ensemble of Tarraco	9	2	29
Renaissance Ensembles of Úbeda and Baeza	7	3	51
Ibiza, biodiversity and culture	11	4	59
Total	114	51	597

Additionally, to ensure the reliability and validity of the data collected, the research team employed a rigorous verification process. This involved cross-referencing information provided by tourism authorities with data obtained directly from tourism companies operating within the historical destinations of the cities. Specifically, the focus was on hotels located within the boundaries of the heritage sites. By triangulating data from multiple sources, including official records and firsthand accounts from industry insiders, the research team aimed to minimize discrepancies and inaccuracies in the dataset.

This multifaceted approach to data collection and verification underscores the commitment to obtaining high-quality and accurate information regarding the hotel occupancy within WHS in Spain. By leveraging both official channels and direct industry engagement, the research team sought to ensure a comprehensive understanding of the hospitality infrastructure within these culturally significant areas. This meticulous methodology not only enhances the reliability of the findings but also contributes to a more nuanced analysis of the relationship between tourism development and heritage conservation in Spain’s World Heritage Sites.

To bridge the gap between the indicators identified through the literature review and the specific circumstances faced by employees in the context of this study, the research team undertook a series of targeted actions. Recognizing the importance of tailoring the indicators to the scope of the research, three focus groups were organized (Sánchez-Oro Sánchez et al., 2021), each comprising a total of 27 hotel employees. These focus groups served as invaluable platforms for gathering insights directly from those immersed in the daily operations and experiences within the hotel industry.

In the initial focus group session, the research team provided a comprehensive overview of the research topic, elucidating its significance and relevance within the context of the hospitality sector. By establishing a shared understanding of the research objectives and themes, participants were primed to engage meaningfully in subsequent discussions.

In the subsequent focus group, participants were invited to express their perspectives and concerns regarding the content and formulation of the research questions. This open dialogue facilitated the refinement of the indicators, ensuring their alignment with the realities and nuances of the hotel employees’ experiences. Feedback garnered from this session served as a valuable guide for enhancing the clarity and relevance of the research instruments.

The culmination of the focus group series involved the recording of interviews centered around the formulated hypotheses (Robina-Ramírez et al., 2021). This phase provided a deeper exploration of the themes and hypotheses under investigation, allowing for rich insights to be captured directly from the voices of hotel employees. Through probing questions and attentive listening, the research team gained nuanced understandings of the intricacies and dynamics at play within the hotel workplace environment.

By integrating the perspectives and inputs gathered from these focus group sessions and interviews, the research team was able to refine and contextualize the identified indicators, ensuring their applicability and resonance within the specific domain of hotel employee experiences. This iterative process of adaptation and refinement not only bolstered the validity and reliability of the research findings but also fostered a deeper appreciation for the complexities inherent in the relationship between employees and the topics under scrutiny.

### 3.2 Measurement model

**Figure 1:**
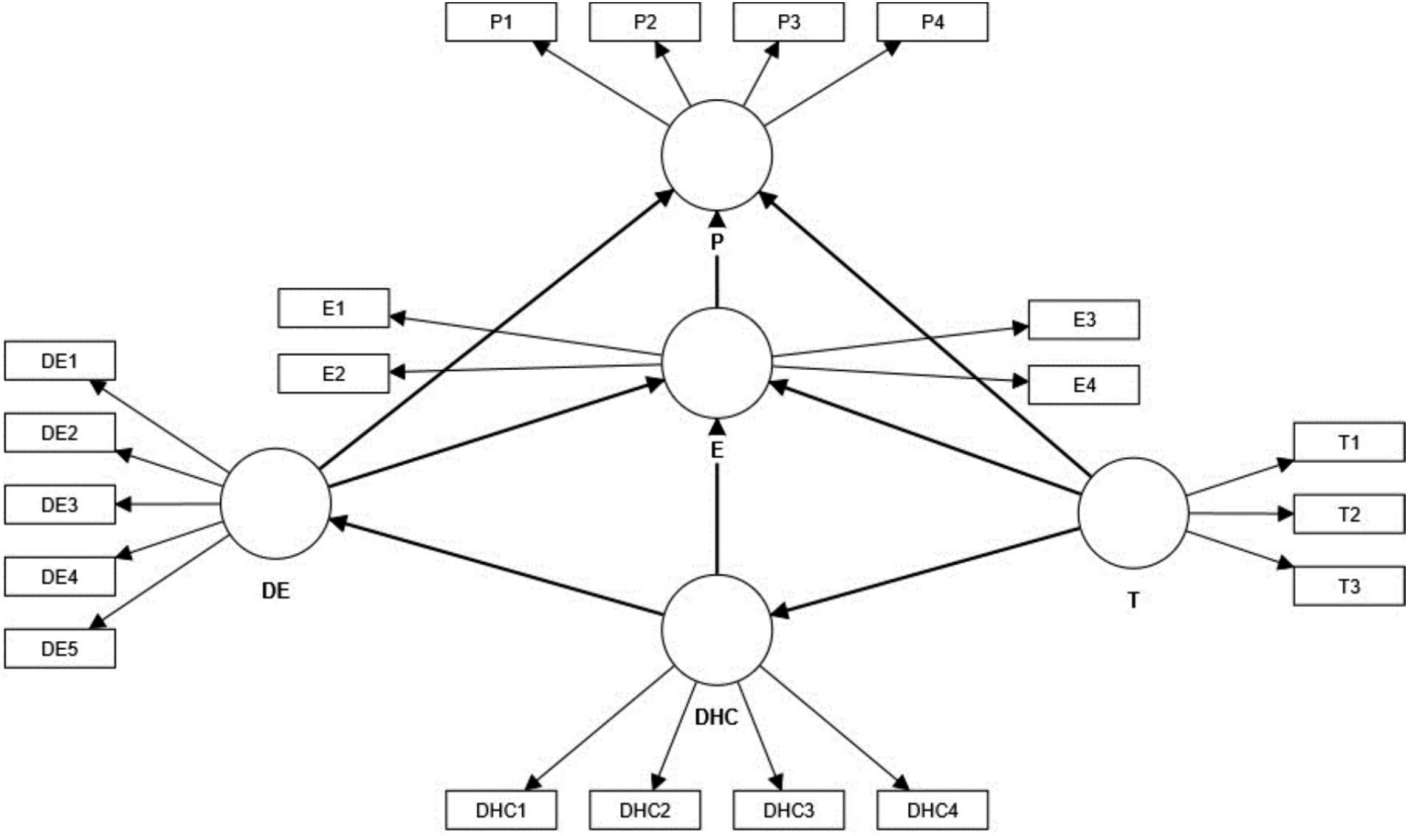
Model.

The five variables analysed in the literature were taken into account for the elaboration of the model. The model presented in Figure 1 shows the indicators that were finally accepted, discarding those that were not significant.

### 3.3 Data processing

Elaborating on variables such as cultural dynamization (DHC), emotions (E), education (DE), and technology (T) in relation to their impact on socio-cultural productivity (P) within hotels at World Heritage Sites (WHS) requires a multifaceted approach that integrates theoretical frameworks, empirical evidence, and advanced statistical analysis.

Firstly, a comprehensive understanding of each variable’s conceptualization and significance within the context of WHS is essential. DHC encompasses initiatives aimed at revitalizing cultural heritage, fostering community engagement, and promoting cultural sustainability, while E relates to the emotional responses of visitors, residents, and stakeholders, shaping their experiences and perceptions within WHS. DE involves educational programs and interpretive materials aimed at enhancing cultural literacy and heritage awareness, while T encompasses digital technologies and multimedia tools used for heritage interpretation and visitor engagement.

By synthesizing theoretical insights with empirical studies and case examples, the teamwork has assess the impact of these variables on socio-cultural productivity within WHS, considering factors such as visitor satisfaction, community cohesion, and heritage conservation efforts.

Utilizing advanced statistical analysis tools like Smart-PLS 4 allows for the calculation of R^2^ values to quantify the variance explained by the model, providing valuable insights into the relative importance and effectiveness of each variable in predicting socio-cultural productivity outcomes within WHS.

This integrated approach facilitates a nuanced understanding of the complex interplay between cultural dynamics, emotional experiences, educational initiatives, technological innovations, and socio-cultural outcomes within the unique context of World Heritage Sites.

Partial least square structural equation modelling (PLS-SEM), coupled with SmartPLS 4, provides tools for gauging the model’s capacity to predict endogenous constructs (Hult et al., 2018). SEM techniques, including the PLS methodology, have gained popularity in analysing tourists’ motivations due to several reasons that make them more suitable than traditional covariance-based SEM techniques in this context (Hair et al., 2011). Tourists’ motivations are a complex and multi-faceted aspect of behavioural research in the field of tourism and hospitality.

## 4 Results

### 4.1 Demographics variables

The demographic analysis explains that of the 597 workers, 58% were women, 68% were under 35, 49% were married and 30% were single.

### 4.2 Measurement model assessment

The evaluation of a PLS-SEM reflective measurement model is a rigorous process that requires to ensure the reliability and validity the internal and external consistency of the model. External loadings represent the strength of the relationship between each latent variable and its indicators, whose value should be >0.7 (Carmines and Zeller, 1979). A considerable number of items were discarded (see Table 3).

**Table 3:** Loadings.

	DE	DHC	E	P	T
DE1	0,867				
DE2	0,878				
DE3	0,817				
DE4	0,831				
DE5	0,890				
DE6	0,601				
DHC1		0,906			
DHC2		0,886			
DHC3		0,804			
DHC4		0,884			
DHC5		0,399			
DHC6		0,502			
E1			0,713		
E2			0,764		
E3			0,821		
E4			0,731		
P1				0,857	
P2				0,847	
P3				0,882	
P4				0,805	
T1					0,896
T2					0,851
T3					0,909
T4					0,445

To study the validity and reliability, we analysed the parameters listed in Table 4. The values range from 0 to 1 (Hair et al., 2011). All conditions are met.

**Table 4:** Reliability and validity.

	Cronbach’s alpha	Composite reliability (rho_a)	Composite reliability (rho_c)	Average variance extracted (AVE)
DE	0,910	0,919	0,933	0,735
DHC	0,894	0,904	0,926	0,759
E	0,756	0,780	0,844	0,575
P	0,870	0,875	0,911	0,719
T	0,862	0,868	0,916	0,784

It is important to note that the Fornell-Larcker criterion only assesses discriminant validity indirectly, by comparing the average variance extracted and correlations (Sarstedt et al., 2011). This may affect the discriminant validity (see Table 5).

**Table 5:** Fornell-Larcker criterion.

	DE	DHC	E	P	T
DE	0,857				
DHC	0,679	0,871			
E	0,412	0,558	0,758		
P	0,660	0,672	0,673	0,848	
T	0,704	0,668	0,384	0,623	0,885

The heterotrait-monotrait ratio of correlations (HTMT) was proposed by Henseler (2021) as an alternative to the Fornell-Larcker criterion for assessing discriminant validity in PLS-SEM. The HTMT is based on comparing correlations between latent variables with indicators of each latent variable (Dash and Paul, 2021). If the HTMT<0.90, there is discriminant validity between the two latent variables (see Table 6).

**Table 6:** HTMT.

	DE	DHC	E	P	T
DE					
DHC	0,737				
E	0,463	0,660			
P	0,741	0,757	0,805		
T	0,799	0,751	0,442	0,721	

### 4.3 Structural model assessment

PLS-SEM is a versatile statistical approach that accommodates the nuances of complex models and latent constructs. PLS-SEM thrives in the realm of relationships, enabling researchers to dissect causal connections and indirect effects. Hypothesis formulation is a foundational step in the research process, guiding investigations and providing a roadmap for analysis. In hypothesis testing, *p*-values and significant t-test are recommended (Cepeda-Carrion et al., 2019). Six out of eight hypothesis are significant (see Table 7).

**Table 7:** Hypotheses.

	Original sample (O)	2.5%	97.5%	T statistics (|O/STDEV|)	*p* values
H1: E - > P	0,455	0,399	0,508	16,321	0,000
H2: DHC - > E	0,520	0,438	0,605	12,146	0,000
H3: DHC - > DE	0,674	0,623	0,726	25,431	0,000
H4: DE - > E	0,066	-0,055	0,188	1,070	0,285
H5: DE - > P	0,312	0,238	0,391	8,152	0,000
H6: T - > DHC	0,667	0,620	0,711	28,511	0,000
H7: T - > E	-0,010	-0,120	0,096	0,181	0,856
H8: T - > P	0,231	0,156	0,306	6,013	0,000

According to Hair et al. (2022) and Henseler (2021) parametric criteria of T-Student (T statistics) and *p* values, allow to say that only null hypotheses 4 and 7 are fulfilled since they present a T-Student value <1.96 and a *p* value >0.05, the bootstrap confidence interval also allows testing whether a path coefficient is significantly different from zero. The confidence interval provides information on the stability of the estimated coefficient by offering a range of plausible population values for the parameter dependent on the variation in the data and the sample size. This implies that educational dynamization does not directly affect employee emotions if the educational initiatives implemented in the workplace primarily focus on skill development or knowledge enhancement rather than emotional regulation.

A fundamental concept within PLS-SEM is the coefficient of determination, (R^2^), which represents the proportion of variance explained in the endogenous constructs. The values for this model were found to be 0.67, 0.33 and 0.10, substantial, moderate, and weak, respectively (Chin, 1998; Table 8).

**Table 8:** R-square.

DE	0,455
DHC	0,446
E	0,313
P	0,656

To measure the fit of the model, several indicators were set. (1) The standardised root mean square residual (SRMR) measures the discrepancy between the observed correlation matrices and the correlation matrices estimated by the model (Dash and Paul, 2021). A SRMR value of less than 0.08 indicates a good model fit. In this case, 0.073 is accepted. (2) The Squared Euclidean distance (d_ULS) and the Geodesic distance (d_G) evaluate the discrepancy between the observed and predicted covariance matrices. (3) The chi-square (χ^2^) compares the observed covariance matrix with the covariance matrix implied by the model. (4) The normed fit index (NFI) values closer to 1 indicating a better fit (see Table 9).

**Table 9:** Saturated model.

SRMR	0,075
d_ULS	1,170
d_G	0,655
Chi-square	2,306,259
NFI	0,757

## 5 Discussion

According to our analysis, the majority of the hypotheses, specifically six out of eight, were validated, with H6 and H3 standing out as particularly significant. H6 suggests a direct influence of technology (T) on historical and cultural dynamisation (DHC) (H6: T - > DHC, β = 0.667; T = 28.511; *p*-value = 0.000). Employees highlighted how technological advancements introduce new organizational dynamics, consequently enhancing socio-cultural productivity by facilitating better planning of experiences in urban destinations (Graziano and Privitera, 2020). Moreover, technology significantly impacts firm productivity at heritage sites (H8: T - > P, β = 0.231; T = 6.013; *p*-value = 0.000), enabling improved visualization of assets within the service delivery chain (Moli, 2011; Al-Hammadi, 2022) and enhancing visitor perception and service configuration at historical places (McCamley, 2016; Cheng and Chen, 2022).

In addition, in traditional areas, technology plays a vital role in enhancing socio-cultural productivity by facilitating community engagement around heritage (Ciolfi et al., 2015; Yan and Wall, 2022) through web-based cultural heritage initiatives (Giglitto, 2017). This favours the cultural and historical dynamics that shape heritage sites by strengthening physical links to the past, as well as their cultural identity (Rouhi, 2017; Cheng and Chen, 2022) and values that have been passed down through the generations (Vecco, 2010).

Furthermore, historical and cultural dynamisation (DHC) positively influences education (DE) (H3: DHC - > DE, β = 0.667; T = 28.511; *p*-value = 0.000). Educational revitalisation generate tourism more responsible for their conservation (Leslie, 2012; Rasoolimanesh et al., 2021) connecting with their own history and cultural identity (Timothy, 2011; Rasoolimanesh et al., 2022). These programmes are designed to gain a comprehensive understanding of the significance of the site, as well as a more enriching and meaningful view of the visit (Fyall et al., 2020).

Emotions also play an essential role in directly improving the sociocultural productivity. As H1 indicates, emotions (E) directly impact productivity (P): E - > P (β = 0.455; T = 16.321; *p*-value = 0.000). Employees experience a range of emotions when visiting other heritage places, such as a sense of awe and reverence for the people who built and inhabited these places (Salemink et al., 2020), beauty, joy (Nisbet and Zelenski, 2011), empathy and connection (Lang et al., 2023). Heritage richness also evokes feelings of belonging and pride (Butler et al., 2022) or nostalgia from the collective memories embedded in architecture, streets and landmarks (Prayag and Del Chiappa, 2023). According to Williams (2006), the emotions generated by positive experiences lead visitors to repeat their visit and to suggest the destination to others which positively affect the sociocultural productivity. These emotions not only influence the tourist’s decision to return, but also allow them to maintain the experience over time by returning repeatedly to remember memorable actions (Tung and Ritchie, 2011; Abd Aziz et al., 2020).

As reflected in H2, historical and cultural dynamisation (DHC) also influence emotions (E) [DHC - > E (β = 0.520; T = 12.146; *p*-value = 0.000)]. The recreation of the past through historical and cultural revitalisation not only brings a sense of continuity and shared identity (Rouhi, 2017; Cheng and Chen, 2022), but also creates a kind of connection that allows for a greater appreciation of the value of historical sites. That recreation generates a range of positive emotions for both visitors and site workers which invite visitors to return to the ancient sites (Van Dijk and Kirk, 2008).

The model presented has a high explanatory power (P: R^2^ = 0.656). We can say that productivity is “moderately” explained by DE = 0.455, DHC = 0.446 and E = 0.313. According to Table 10, if we focus exclusively on the values that define the high significance of P, we observe that 65.6% is explained by 30.6% by the emotions (E) derived from the experiences obtained by employees when they visit other heritage sites, 20.6% by the learning capacity (DE) and 14.5% by technology (T).

**Table 10:** Explained variance of the model.

	Adjusted R^2^	Direct Effect	Correlation	Variance explained
**P**	0,656			
E		0,455	0,672	0,306
DE		0,312	0,661	0,206
T		0,231	0,626	0,145
**E**	**0,313**			**0,656**
DE		0,066	0,41	0,027
DHC		0,52	0,558	0,290
T		-0,01	0,384	-0,004
**DHC**	0,446			**0,313**
T		0,667	0,667	**0,446**
**DE**	0,455			
DHC		0,674	0,674	**0,455**

## 6 Conclusion

### 6.1 Theoretical implications

On the basis of the studies analysed and in accordance with the results obtained, we can highlight some theoretical implications:

First, for a high number of the employees who participated in this study, the sociocultural productivity of heritage sites is connected not only with physical heritage but also with cultural, historical, scientific or natural heritage because of its unique and significant value (Flores de León et al., 2020). This intangible heritage enables the development of cultural, historical (Su, 2020), educational and learning activities.

Second, more than half of the improvements in the sociocultural productivity of heritage sites are explained by the emotions of visitors to heritage places. Fostering positive emotional connections not only contributes to improved visitor expectations for these environments, but also creates a virtuous circle that benefits both visitors and site workers (Van Dijk and Kirk, 2008).

Third, technology currently plays an essential role in cultural-historical dynamization. Advances in technology also allow for the pursuit of greater community participation, which also facilitates the promotion of dialogue between visitors (Simon, 2010; Mugobi and Mlozi, 2021) and their dissemination and access impacting positively to the sociocultural productivity at the historical sites (Affleck and Kvan, 2008; Liang et al., 2021).

Fourth, emotions are generated from historical and cultural dynamisation. This evidence comes from the affirmation of H3 and rejection of H4 and H7. Historical-cultural dynamisation becomes the primary reason for the generation of emotions, as explained by the third hypothesis. Emotions are generated not only by the ability of heritage sites to connect with the past through their architectural wonders and narratives (Lowenthal, 2005; Zhu et al., 2022; Arumugam et al., 2023), but also because they help to build the identity of visitors who come to these places to better understand their history (Rouhi, 2017; Cheng and Chen, 2022) from the traditions and values passed on through the generations (Vecco, 2010).

### 6.2 Practical implications

First of all, it is necessary to highlight the importance of the proposed model as a tool for improving the productivity of heritage sites based on the experience of hotel employees. The fact that they work in these historic sites makes them qualified agents in defining the main factors that affect the productivity of these sites. This information may be of great interest to the tourism authorities and businesses residing in such historic environments.

Second, with the growth in technological advances, cultural institutions are beginning to orient the promotion and productivity of heritage places towards participatory design approaches that encourage dialogue with their visitors, adopting practices that are more focused on cultural demand (Simon, 2010; Mugobi and Mlozi, 2021). This also allows for the better management of both visitor experiences and the emotions generated from them (Ciolfi et al., 2016; Yan and Wall, 2022) through better control over cultural content to decide what, where and how to share it with visitors (Giaccardi, 2012). All this activity allows for the better collection, management or display of cultural heritage (Giglitto, 2017), which has an impact on improving its productivity.

Third, the improvement of educational dynamics has a positive effect on productivity, as stated in the fifth hypothesis (SD - > P, β = −0.312; T = 8.152; *p*-value = 0.000). This enhancement requires fostering a deeper understanding of these sites among local visitors (Leslie, 2012; Rasoolimanesh et al., 2021) through educational training programmes designed by tourism authorities (Ritchie, 2003; Abd Aziz et al., 2020). The aim of these cultural actions is to provide a better connection with the historical and cultural attractions of heritage sites (Timothy, 2011; Rasoolimanesh et al., 2022), places that help to deepen the visitor’s understanding of the reality of the past by fostering emotions such as a sense of belonging and pride.

### 6.3 Limitations and future directions

This study has several limitations. First, the data were collected only from tourism enterprises and not from the authorities. If this investigation had covered both perspectives, it would have been able to provide a more complete picture. The current political situation in Spain has made it impossible to implement this second option, since many of the Directorates-General of Tourism still do not have a person appointed to gather data. This limitation suggests a future line of research that the research team will address in the coming months.

Future studies could include the proposal of suggestions for the incorporation of technology based on an increase in their productivity aimed at both ICOMOS bodies and the tourism authorities that manage heritage sites for the promotion of historical places.

## Data availability statement

The original contributions presented in the study are included in the article/supplementary material, further inquiries can be directed to the corresponding author.

## Ethics statement

Ethical review and approval was not required for the study on human participants in accordance with the local legislation and institutional requirements. Written informed consent from the patients/ participants or patients/participants' legal guardian/next of kin was not required to participate in this study in accordance with the national legislation and the institutional requirements.

## Author contributions

MM-L: Writing – review & editing, Writing – original draft, Visualization, Validation, Supervision, Software, Resources, Project administration, Methodology, Investigation, Funding acquisition, Formal analysis, Data curation, Conceptualization. AL-S: Writing – review & editing, Writing – original draft, Visualization, Validation, Supervision, Software, Resources, Project administration, Methodology, Investigation, Funding acquisition, Formal analysis, Data curation, Conceptualization. ÁP: Writing – review & editing, Writing – original draft, Visualization, Validation, Supervision, Software, Resources, Project administration, Methodology, Investigation, Funding acquisition, Formal analysis, Data curation, Conceptualization. RR: Visualization, Validation, Supervision, Software, Resources, Project administration, Methodology, Investigation, Funding acquisition, Formal analysis, Data curation, Conceptualization, Writing – review & editing, Writing – original draft. LM-L: Visualization, Validation, Supervision, Software, Resources, Project administration, Methodology, Investigation, Funding acquisition, Formal analysis, Data curation, Conceptualization, Writing – review & editing, Writing – original draft.
