# Correction: Do intangible factors enhance sociocultural productivity and economy in world heritage sites?

**DOI:** 10.3389/fpsyg.2025.1672701

**Published:** 2025-08-05

**Authors:** María Martín-Lucas, Ana Leal-Solís, Ángel Pizarro Polo, Rafael Robina Ramírez, Libertad Moreno-Luna

**Affiliations:** ^1^Department of Business Management and Sociology, Universidad de Extremadura, Cáceres, Spain; ^2^Department of Building, Universidad de Extremadura, Cáceres, Spain

**Keywords:** productivity, heritage sites, tourism, emotions, architecture, economy

In the original published article, author María Martín-Lucas was erroneously assigned as corresponding author. The correct corresponding author is Rafael Robina Ramírez, rrobina@unex.es.

The original version of this article has been updated.

