# Correction: Do intangible factors enhance sociocultural productivity and economy in world heritage sites?

**DOI:** 10.3389/fpsyg.2025.1688151

**Published:** 2025-09-15

**Authors:** María Martín-Lucas, Ana Leal-Solís, Ángel Pizarro Polo, Rafael Robina Ramírez, Libertad Moreno-Luna

**Affiliations:** ^1^Department of Business Management and Sociology, Universidad de Extremadura, Cáceres, Spain; ^2^Department of Building, Universidad de Extremadura, Cáceres, Spain

**Keywords:** productivity, heritage sites, tourism, emotions, architecture, economy

In the original published article, an incorrect funding statement was provided. The correct funders are the European Union, the European Regional Development Fund and the Regional Government of Extremadura. Instead of “The author(s) declare that no financial support was received for the research, authorship, and/or publication of this article”, the correct funding statement should be written as shown below.

“The author(s) declare that financial support was received for the research and/or publication of this article. This publication has been 85% co-financed by the European Union, the European Regional Development Fund, and the Regional Government of Extremadura. Managing Authority: Ministry of Finance. File No.: GR24083.”

The original version of this article has been updated.

